# Nuclear factor-κB activation in perihematomal brain tissue correlates with outcome in patients with intracerebral hemorrhage

**DOI:** 10.1186/s12974-015-0277-9

**Published:** 2015-03-15

**Authors:** Ze-Li Zhang, Yu-Guang Liu, Qi-Bing Huang, Hong-Wei Wang, Yan Song, Zhen-Kuan Xu, Feng Li

**Affiliations:** Department of Emergency Surgery, Qilu Hospital of Shandong University, No. 107 Wenhuaxi Road, 250012 Jinan, Shandong Province People’s Republic of China; Department of Neurosurgery, Qilu Hospital of Shandong University and Brain Science Research Institute of Shandong University, No. 107 Wenhuaxi Road, 250012 Jinan, Shandong Province People’s Republic of China

**Keywords:** NF-κB, Intracerebral hemorrhage, Outcome, Predictor, Patient

## Abstract

**Background:**

Nuclear factor-κB (NF-κB) plays an important role in the inflammatory response after intracerebral hemorrhage (ICH). We therefore proposed that NF-κB activation in perihematomal brain tissue might correlate with clinical outcome in patients with ICH. To confirm this, we studied clinical data of 45 patients with ICH and NF-κB activation in perihematomal brain tissue and analyzed predictors of clinical outcome as well as the predictive value of NF-κB activation.

**Methods:**

Forty-five patients with spontaneous basal ganglia hemorrhage were prospectively investigated. The clinical data were collected, which include demographics, alcohol and tobacco abuse, stroke risk factors, neuroimaging variables at presentation, Glasgow Coma Scale (GCS) at admission, number of days in hospital, mechanical ventilation, pneumonia, and outcome. Clinical outcome was assessed by the modified Rankin Scale at 6 months after ICH. Perihematomal brain tissue was collected, and NF-κB activation was detected using immunohistochemistry. Univariate analysis and multivariate logistic regression analysis were performed to determine predictors of the poor outcome.

**Results:**

Immunohistochemical detection showed that NF-κB p65 was expressed in the nuclei of neurons and glial cells in all patients. The number of nuclear NF-κB p65-positive cells was 54 ± 21. Six months after ICH, 18 (40%) patients achieved a favorable functional outcome (mRS ≤ 3) while 27 (60%) had a poor functional outcome (mRS 4 to 6). In univariate analysis, predictors of poor functional outcome were lower GCS score on admission (*P* = 0.004), larger hematoma volume (*P* = 0.004), intraventricular extension (*P* = 0.047), midline shift (*P* = 0.005), NF-κB activation (*P* < 0.0001), mechanical ventilation (*P* = 0.018), and co-morbidity with pneumonia (*P* = 0.002). In multivariate logistic regression analysis, NF-κB activation was the only independent predictor of poor outcome at 6 months after ICH.

**Conclusions:**

NF-κB activation is closely related to clinical outcome 6 months after ICH in humans. Therefore, it could be useful to predict prognosis of ICH accurately and should be further evaluated as a target for therapeutic strategies of ICH in the future.

## Introduction

Primary intracerebral hemorrhage (ICH) is still a frequent form of cerebrovascular diseases despite improved control of hypertension. It accounts for 30% of all cases of stroke in China, approximately twice higher than that in the West, and is one of the leading causes of stroke-related mortality and morbidity worldwide [1-3]. Functional outcome in survivors is also poor with fewer than 20% being independent at 6 months [4]. Previous studies have shown that the prognosis of patients with ICH is affected by series of factors, such as age, high blood pressure, hematoma volume, Glasgow Coma Scale (GCS) score on admission, stroke risk factors, underlying disease, leukocyte counts, neuroimaging, operation, complications, and so on [5-11], so it is always difficult for us to predict the outcome accurately.

A series of pathophysiological changes in brain tissue arises after ICH [12]. Previous studies revealed that a large number of inflammatory cells surround the hematoma in the rat model of ICH and that the inflammatory response is an important mechanism of secondary brain damage after ICH [13,14]. Nuclear factor-κB (NF-κB) has been recognized as a critical regulator of inflammatory responses since its discovery [15]. In unstimulated cells, inactive NF-κB is sequestered in the cytoplasm by inhibitory protein IκB. NF-κB can be activated by a wide array of factors such as thrombin, tumor necrosis factor-α (TNF-α), interleukin-1 (IL-1), oxidative stress, and growth factors [16]. Then, the free NF-κB rapidly migrates into the nucleus, binds to DNA, and promotes the transcription of genes for the release of inflammatory substances. So NF-κB plays an important role in the inflammatory response after ICH [17].

Given the important role played by NF-κB in the secondary brain damage after ICH, we imagine that NF-κB activation in perihematomal brain tissue was closely related to the clinical outcome of patients with ICH. In other words, NF-κB could be used to predict the clinical outcome. In order to confirm this, we collected 45 patients with basal ganglia hemorrhage, studied the clinical data and perihematomal brain tissue, and analyzed the predictors of the clinical outcome as well as the predicting value of NF-κB activation.

## Materials and methods

### Study population

All patients with spontaneous basal ganglia hemorrhage admitted to the Emergency Neurosurgery Department of Shandong University Qilu Hospital from October 2011 to August 2013 were screened for this study. Inclusion criteria were time from symptom onset to specimen collection 6 to 12 h, hematoma volume 30 to 90 ml, and hematoma evacuation operation conducted along the non-functional cortex. The intracerebral hemorrhage volume was measured using the ABC/2 method according to the CT scanning [18]. Exclusion criteria were rebleeding, secondary ICH (such as head trauma, aneurysm, vascular malformation, hemorrhagic infarction, cerebral vein and sinus thrombosis, tumor, anticoagulant, blood thinners, or coagulopathy-related hemorrhage), history that may affect the study (such as bleeding, inflammation, trauma, surgery), use of drugs that affect the immune system (ibuprofen, hormones, illicit drug, cocaine or other stimulants, and so on), presence of underlying diseases within the previous month, or refusal of participation.

### Ethics approval

The study protocol was approved by the Ethics Committee of Qilu Hospital. All patients’ families received a comprehensive description of the study and gave written informed consent for their relatives’ participation. The specimen for the study was waste brain tissue discarded during surgery, and the collection process did not cause any additional damage to the patient.

### Clinical data and specimen collection

Clinical data of patients were collected on admission or during hospitalization. The variables include demographics (age and gender), alcohol and tobacco abuse, a detailed history of stroke risk factors (hypertension, diabetes mellitus, coronary heart disease, and chronic obstructive pulmonary diseases (COPD)), neuroimaging variables at presentation (hematoma volume, intraventricular extension, midline shift, hydrocephalus, brain edema), GCS on admission, number of days in hospital, mechanical ventilation, pneumonia, and outcome. The midline shift was determined by the distance between midline and septum pellucidum according to the CT scanning.

The proximity to the ICH is a key factor in the inflammatory response. Therefore, the specimen (no less than 0.5 cm^3^ per patient) was collected according to the standard that the distance between specimen and hematoma was 1 cm. In order to reduce the traction injury to the adjacent brain tissue, cortical fistula was made according to the ICH location during operation. Specimen was collected in this process by the same surgeon, and the distance between specimen and hematoma can be measured directly. So the samples were collected in the same way each time. The brain tissue was quickly fixed with 10% formalin and embedded in wax for immunohistochemistry (IHC) detection of NF-κB activation.

### Detection of NF-κB activation

The activation of NF-κB was detected by IHC. The tissue sections (4 μm thick) were dewaxed, rehydrated, rinsed with distilled water and phosphate-buffered saline (PBS), repaired with EDTA, quenched with 3% H_2_O_2_, exposed to primary antibody (anti-NF-κB p65 antibody, B7162 rabbit polyclonal, ANBO, USA), and incubated at 4°C overnight. Sections were then washed with PBS, incubated in polymer helper for 25 min at room temperature, washed again, and incubated with non-biotin rabbit hypersensitivity two-step secondary antibody (PV-9001, GBI, USA) for 25 min at room temperature. Finally, the sections were stained with diaminobenzidine-H_2_O_2_ solution, washed, dehydrated in graded ethanol, immersed in xylene, and covered with a coverslip. In order to identify the cell type of nucleus NF-κB positive cells (neurons or glial cells), double-labeled IHC was performed on all of the 45 specimens, using the primary antibody (anti-NF-κB p65 antibody, B7162 rabbit polyclonal, ANBO, USA; anti-GFAP antibody, TA500336 mouse monoclonal, ZSGB-BIO, CHN; anti-NSE antibody, ZM-0203 mouse monoclonal, ZSGB-BIO, CHN) and double staining kit (DS-0005, ZSGB-BIO, CHN). The double-labeled IHC was performed according to the instructions of the double staining kit.

The slices were observed under the multi-head microscope by five professors of pathology in a blinded fashion. A total of five no-repeat fields (×400 high magnification) were randomly selected, the nucleus NF-κB positive cells were identified, and the numbers of positive cells in the five fields were added up as the result. The numbers of positive cells recorded by the five pathologists were consistent.

### Functional outcome assessment

Clinical outcome was assessed by modified Rankin Scale (mRS) at 6 months after ICH. The follow-up was made by telephone interview or face-to-face assessment. In this study, the patients were relatively serious due to the hematoma volume, and three points can be considered as a good prognosis. Therefore, poor clinical outcome was defined as mRS ≥ 4 assessed at the 6-month follow-up.

### Statistical analysis

The normally and non-normally distributed continuous variables were expressed as mean ± standard derivation and median (IQR), respectively. In univariate analysis, normally distributed continuous variables were analyzed with Student’s *t*-test, non-normally distributed continuous variables were analyzed with Mann–Whitney *U* test, and categorical variables were analyzed with chi-square test. Stepwise forward logistic regression was used to determine independent predictors for poor functional outcome at 6 months after ICH. All tests were two-tailed, and statistical significance was determined at *α* level of 0.05. Statistical analysis and charting were performed using SPSS 19.0 and Excel 2003.

## Results

### Clinical data and NF-κB activation

The clinical data and NF-κB activation are listed in Table 1. The number of patients with 30 to 90 ml basal ganglia ICH was 419 in total, and 92 (22.0%) refused surgery because of serious underlying disease or some other personal reasons (such as economic reasons and so on). All of the others (327, 78.0%) were treated with surgery in our department. Among patients with an ICH size of 30 to 90 ml that underwent surgical decompression, a total of 45 patients met our study’s inclusion criteria, with an age of 53.87 ± 10.78 years (range 35 to 77 years), 29 males and 16 females. Thirty-seven (82.2%) had one or more underlying diseases: 26 (57.8%) had hypertension, 11 (24.4%) diabetes mellitus, 5 (11.1%) COPD, and 11 (24.4%) coronary artery diseases. Fourteen (31.1%) were smokers, and 11 (24.4%) were drinkers. The GCS score on admission was 5 to 13.

**Table 1:** **Predictive value of the characteristics on univariate analysis**

**Characteristics**	**Total (** ***n*** **= 45)**	**Good outcome (** ***n*** **= 18)**	**Poor outcome (** ***n*** **= 27)**	**Odds ratio (95% CI)**	***P*** **value**
Demographics					
Male sex	29 (64.4)	10 (55.6)	19 (70.4)	1.900 (0.548 to 6.590)	0.309^a^
Age, years	53.87 ± 10.78	51.50 ± 10.40	55.44 ± 10.93		0.233^b^
GCS score on admission	9 (4)	11 (3.5)	9 (2)		0.004^c^
Risk factors					
Smoking	14 (31.1)	5 (27.8)	9 (33.3)	1.300 (0.352 to 4.796)	0.693^a^
Alcohol abuse	11 (24.4)	5 (27.8)	6 (22.2)	0.743 (0.188 to 2.934)	0.671^a^
Hypertension	26 (57.8)	10 (55.6)	16 (59.3)	1.164 (0.348 to 3.885)	0.805^a^
Diabetes mellitus	11 (24.4)	4 (22.2)	8 (29.6)	1.474 (0.369 to 5.885)	0.582^a^
Coronary heart disease	11 (24.4)	3 (16.7)	8 (29.6)	2.105 (0.475 to 9.338)	0.322^a^
COPD	5 (11.1)	1 (5.6)	4 (14.8)	2.957 (0.303 to 28.882)	0.333^a^
Radiologic variables					
Hematoma volume, ml	59.44 ± 14.26	52.17 ± 13.10	64.30 ± 13.06		0.004^b^
Intraventricular extension	18 (40.0)	4 (22.2)	14 (51.9)	3.769 (0.984 to 14.443)	0.047^a^
Hydrocephalus	10 (22.2)	2 (11.1)	8 (29.6)	3.368 (0.624 to 18.185)	0.143^a^
Midline shift ≥1 cm	24 (53.3)	5 (27.8)	19 (70.4)	6.175 (1.647 to 23.148)	0.005^a^
Brain edema	13 (28.9)	3 (16.7)	10 (37.0)	2.941 (0.680 to 12.730)	0.140^a^
Hospitalizations					
Mechanical ventilation	14 (31.1)	2 (11.1)	12 (44.4)	6.400 (1.224 to 33.482)	0.018^a^
Pneumonia	20 (44.4)	3 (16.7)	17 (63.0)	8.500 (1.964 to 36.790)	0.002^a^
NF-κB activation	54.38 ± 20.97	37.11 ± 16.51	65.89 ± 14.91	2.929 (1.616 to 5.311)^d^	0^b^

^a^Chi-square test for categorical variables, data was expressed as *n* (%); ^b^Student’s *t*-test for normally distributed continuous variables, data was expressed as mean ± SD; ^c^Mann-Whitney *U* test for non-normally distributed continuous variables, data was expressed as median (IQR); ^d^The numbers of nucleus NF-κB p65-positive cells were stratified by 10, and OR (95% CI) was calculated according to the binary logistic regression analysis.

CT scan was performed on all of the 45 patients. The intracerebral hematoma volume was 59.44 ± 14.26 ml. Eighteen (40.0%) had intraventricular extension, 10 (22.2%) had acute hydrocephalus, 13 (28.9%) had perihematoma brain edema, and 24 (53.3%) had midline shift ≥1 cm.

Hematoma evacuation operation was performed on all patients along the non-functional cortex, while ventricular drainage was performed on 16 (35.6%) and craniectomy on 12 (26.7%). Mechanical ventilation was required in 14 (31.1%) patients, and pneumonia was diagnosed in 20 (44.4%) patients. The body temperature was controlled between 36.0°C and 37.0°C with the help of drugs or physical cooling. Osmotherapy (mannitol or hypertonic saline) was used pre- or post-operation. Mannitol was used according to the clinical manifestations and imaging. Serum sodium was maintained at 145 mmol/l or higher if necessary. The numbers of days in hospital were 18.29 ± 6.89. Do-not-attempt resuscitation or withdrawal-of-care did not exist in all of the 45 patients.

**Figure 1:**
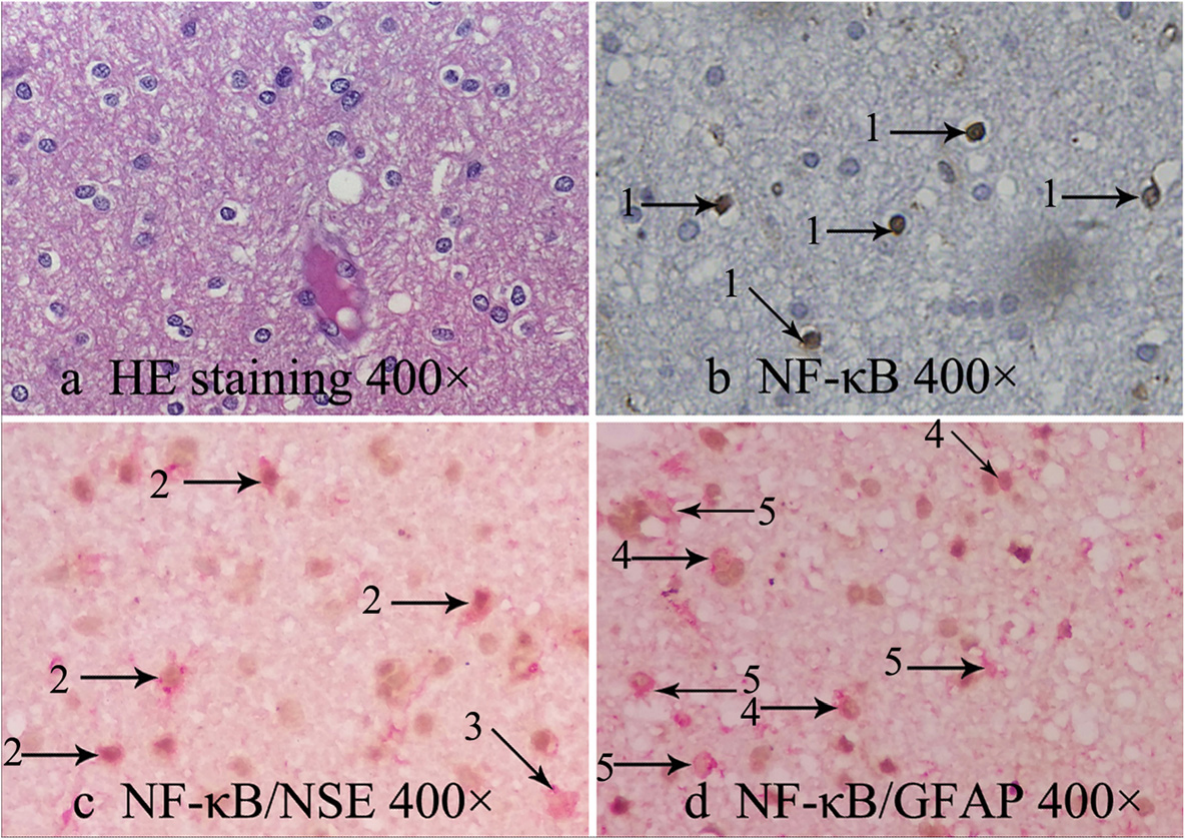
**The microscopic images of NF-κB p65 detected with IHC.** Microscopic images (400×) showed that NF-κB p65 expressed in nucleus of neurons and glial cells. **(a)** HE staining. **(b)** NF-κB p65 detected with IHC. **(c)** NF-κB p65/NSE double-labeled IHC. **(d)** NF-κB p65/GFAP double-labeled IHC. 1 → indicates nucleus NF-κB p65-positive neurons or glial cells, 2 → indicates nucleus NF-κB p65-positive neurons, 3 → indicates nucleus NF-κB p65-negative neurons, 4 → indicates nucleus NF-κB p65-positive glial cells, and 5 → indicates nucleus NF-κB p65-negative glial cells. HE, hematoxylin and eosin; NF-κB, nuclear factor-κB; NSE, neuron-specific enolase; GFAP, glial fibrillary acidic protein.

Immunohistochemical detection showed that NF-κB p65 was expressed in the nucleus of cells in all of the 45 patients (Figure 1), suggesting that NF-κB was activated and migrated into the nucleus. Double-labeled IHC showed that NF-κB p65 was expressed in the nucleus of both neurons and glial cells (Figure 1). The numbers of nucleus NF-κB p65-positive cells ranged from 9 to 95, and the total number was 54.38 ± 20.97.

The numbers of patients mRS scored 0 to 6 at 6 months after ICH were 0, 3 (6.5%), 8 (17.4%), 7 (15.2%), 9 (19.6%), 16 (34.8%), and 2 (4.3%); thus, 18 (40.0%) patients achieved a favorable functional outcome (mRS ≤ 3) while 27 (60.0%) had a poor functional outcome (mRS 4 to 6).

### Predictors of poor outcome at 6 months after ICH

In univariate analysis of the 6-month outcome, predictors of poor functional outcome were lower GCS score on admission (*P* = 0.004), larger hematoma volume (*P* = 0.004), intraventricular extension (*P* = 0.047), midline shift (*P* = 0.005), and NF-κB activation (*P* < 0.0001). In addition, significantly more patients with poor outcome needed mechanical ventilation (*P* = 0.018) and suffered from pneumonia (*P* = 0.002). There was no significant difference in sex, age, all of the researched risk factors, brain edema, or hydrocephalus on admission. Then, the numbers of nucleus NF-κB p65-positive cells were stratified by 10, and the result was 1 case for 0~, 2 for 10 ~ and 20~, 8 for 30~, 5 for 40~, 6 for 50~, 8 for 60 ~ and 70~, 3 for 80~, and 2 for 90~. According to the binary logistic regression analysis, *β* value was 1.075, OR was 2.929, and the 95% CI was 1.616 to 5.311 (Table 1).

**Figure 2:**
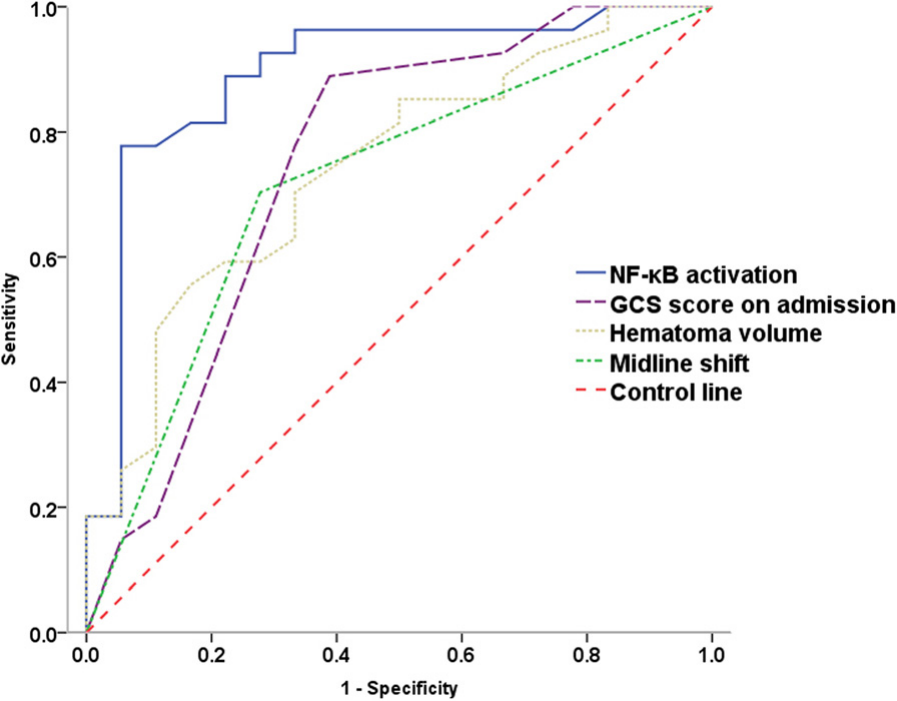
**The ROC curve of predictors to predict the poor clinical outcome.** The AUC of NF-κB activation, GCS score on admission, hematoma volume, and midline shift were 0.893 (95% CI 0.787 to 0.999), 0.748 (95% CI 0.590 to 0.906), 0.744 (95% CI 0.598 to 0.890), and 0.713 (95% CI 0.556 to 0.870), respectively. NF-κB, nuclear factor-κB; GCS, Glasgow Coma Scale.

In the multivariate analysis, the variable entrance cutoff was set as 0.10 according to the results of univariate analysis. Therefore, GCS score on admission, hematoma volume, intraventricular extension, midline shift, and NF-κB activation were selected for multivariate analysis using logistic regression. With stepwise logistic regression, NF-κB activation was the only independent predictor of the 6-month outcome. The prognostic accuracy of NF-κB activation was assessed with ROC curve analysis. The area under curve (AUC) of NF-κB activation was 0.893 (95% CI 0.787 to 0.999), which was higher than that of GCS score on admission (0.748, 95% CI 0.590 to 0.906), hematoma volume (0.744, 95% CI 0.598 to 0.890), and midline shift (0.713, 95% CI 0.556 to 0.870) (Figure 2).

### Relationship between baseline data and NF-κB activation

The relationship between every baseline data and NF-κB activation was analyzed. Continuous variables were analyzed with binary linear regression, and categorical variables were analyzed with one-way ANOVA. The results showed that NF-κB activation was significantly related to GCS score on admission (*P* < 0.0001), hematoma volume (*P* < 0.0001), and midline shift (*P* = 0.006) and not significantly related to sex, age, risk factors, or radiologic variables except midline shift.

## Discussion

The present study examines the relationships among clinical data, NF-κB activation, and the 6-month outcome following primary ICH and produces the following major findings. First, in univariate analysis, GCS score on admission, hematoma volume, intraventricular extension, midline shift, mechanical ventilation, pneumonia, and NF-κB activation were predictors of the 6-month functional outcome. Second, in multivariate analysis, only NF-κB activation was independently associated with the 6-month outcome. Third, on the linear regression analysis, NF-κB activation was closely related to GCS score on admission, hematoma volume, and midline shift.

In this study, GCS score on admission, hematoma volume, intraventricular extension, and midline shift were predictors of the 6-month outcome, and this was consistent with the literature [5-11]. But one thing should be noted is that preoperative brain edema was not related to the 6-month outcome according to the univariate analysis. The reason for this may be that the CT scanning was performed within 10 h after onset of ICH, when the edema just begun and had not reached the peak.

It is interesting that this study first newly identified NF-κB activation as the only independent factor to predict the poor clinical outcome after ICH according to the multivariate analysis. This is mainly because that NF-κB activation is affected by many baseline factors. In the linear regression analysis, NF-κB activation was closed related to GCS score on admission, hematoma volume, and midline shift. Therefore, NF-κB activation might be affected by the combined effect of the three factors and then became the only independent factor.

The close relationship between NF-κB activation and the 6-month outcome is probably due to the function of NF-κB’s downstream factors. NF-κB is a ubiquitous transcription factor and a member of a family of proteins which are critical regulators of a variety of responses, including inflammation [19]. In unstimulated cells, inactive NF-κB is sequestered in the cytoplasm by inhibitory protein IκB, which prevents its translocation to the nucleus. In response to various external pathogenic stimuli, including thrombin, TNF-α, IL-1β, oxidative stress, growth factors, and so on [16,20,21], specific kinases phosphorylate IκB, leading to its proteolysis and dissociation from NF-κB. The free, newly activated NF-κB migrates into the cell nucleus, where it binds to specific NF-κB response elements in the promoters of target genes. This promotes the transcription of genes for the release of series of inflammatory substances, such as TNF-α, IL-1β, induced nitric oxide synthase (iNOS), intercellular adhesion molecule-1 (ICAM-1), and so on. These substances are closely related to the secondary neuronal injuries after ICH, including blood–brain barrier disruption, brain edema, and neuronal cell death [22-29], which lead to poor functional outcome.

The results of this study have two major significances for the treatment of ICH in the future. First, based on these results, NF-κB activation in the perihematomal tissue is closely related to the outcome of ICH patients, so it can be detected to predict the prognosis of ICH accurately. Second and more importantly, these findings offer a potential therapeutic target for patients with ICH. It might be possible to improve the outcome of ICH patients by interfering NF-κB activation. Studies on animal ICH model have confirmed that some measures, such as application of 6-O-acetyl shanzhiside methyl ester, tauroursodeoxycholic acid in 2 h after ICH, and neural stem cell transplantation, could reduce the NF-κB activation [30-32]. Therefore, these measures might be taken to treat human ICH after clinical trials in the future.

There are several limitations in the current study that should be addressed. On one hand, the sample size of this study was relatively small and the patients enrolled were relatively serious, because the brain tissue could only be collected from patients with poor grades, who needed surgery to evacuate the hematoma. The result of this study may not be representative for all ICH patients. Therefore, we will continue collecting more cases in order to obtain a more convincible result. On the other hand, although NF-κB activation in the perihematomal tissue is closely related to the outcome of ICH patients, it is still questionable whether NF-κB activation directly leads to poor functional outcome or NF-κB activation is just a compensatory mechanism to protect the brain tissue from damage. In other words, promoting or inhibiting NF-κB activation to improve the outcome of ICH patients is still unclear. To clarify this issue, we will study the ICH rat model, using gene overexpression, RNA interference, and specific inhibition technology to interfere NF-κB activation at the different stages after ICH, detect the cell death, observe the behavioral changes of rats, and finally clarify the effect of NF-κB activation to the cell death and outcome in different stages. Then, different measures can be performed in different stages to improve the outcome of ICH.

## Conclusion

In summary, in the current study, we found that NF-κB activation was closely related to the 6-month clinical outcome after primary ICH in humans. Therefore, it can be detected to predict the prognosis of ICH accurately. Furthermore, these findings offer a potential therapeutic target for patients with ICH. It might be possible to improve the outcome of ICH patients by interfering NF-κB activation in the future.

## Abbreviations

AUC: area under curve
COPD: chronic obstructive pulmonary diseases
GCS: Glasgow Coma Scale
ICAM-1: intercellular adhesion molecule-1
ICH: intracerebral hemorrhage
IHC: immunohistochemistry
IL-1β: interleukin-1β
iNOS: induced nitric oxide synthase
NF-κB: nuclear factor-κB
NIK: NF-κB-inducing kinase
ROC curve: receiver operating characteristic curve
TNF-α: tumor necrosis factor-α
